# The Short ITS2 Sequence Serves as an Efficient Taxonomic Sequence Tag in Comparison with the Full-Length ITS

**DOI:** 10.1155/2013/741476

**Published:** 2013-01-17

**Authors:** Jianping Han, Yingjie Zhu, Xiaochen Chen, Baoshen Liao, Hui Yao, Jingyuan Song, Shilin Chen, Fanyun Meng

**Affiliations:** ^1^Institute of Medicinal Plant Development, Chinese Academy of Medical Sciences and Peking Union Medical College, Beijing 100094, China; ^2^College of Resources Science & Technology, Beijing Normal University, Beijing 100875, China

## Abstract

An ideal DNA barcoding region should be short enough to be amplified from degraded DNA. In this paper, we discuss the possibility of using a short nuclear DNA sequence as a barcode to identify a wide range of medicinal plant species. First, the PCR and sequencing success rates of ITS and ITS2 were evaluated based entirely on materials from dry medicinal product and herbarium voucher specimens, including some samples collected back to 90 years ago. The results showed that ITS2 could recover 91% while ITS could recover only 23% efficiency of PCR and sequencing by using one pair of primer. Second, 12861 ITS and ITS2 plant sequences were used to compare the identification efficiency of the two regions. Four identification criteria (BLAST, inter- and intradivergence Wilcoxon signed rank tests, and TaxonDNA) were evaluated. Our results supported the hypothesis that ITS2 can be used as a minibarcode to effectively identify species in a wide variety of specimens and medicinal materials.

## 1. Introduction

### 1.1. DNA Barcoding of Degraded DNA Materials

DNA barcoding takes advantage of short standard sequences to discover and identify species [1]. An ideal DNA barcode should be short enough to be amplified from archival specimens using universal primers. The term “minimalist barcode” was first defined by Herbert as a tool to overcome the low PCR efficiency of cytochrome c-oxidase subunit 1 (CO1) in archival animal specimens in museums, and the possibility of identifying animal specimens using a region of approximately 200 bp was discussed. The results of that study showed that minibarcodes can be isolated from different types of specimens, including museum samples, trace tissue samples with degraded DNA and other specimens, from which the acquisition of a full-length barcode (CO1) is not feasible [2]. The amplification of DNA from herbarium specimens is also important for barcoding studies because it is often necessary to confirm the species identification of fresh specimens by comparing their sequences with those of older museum specimens [3]. Additionally, most of the medicinal materials available in the market are dry and have been stored for long periods; thus, it is very difficult to amplify long DNA regions from some of these materials, which prevent the use of DNA barcodes for herb identification.

### 1.2. The Trend of Core Plant DNA Barcodes

The Plant Working Group of the Consortium for the Barcode of Life (CBOL) recommended the use of a combination of *matk* and *rbcL *as a barcode for land plants [4], and internal transcribed spacer (ITS)/internal transcribed spacer 2 (ITS2) was proposed as a supplemental marker for further study. The ITS sequence contains enough variable sites for species identification in many samples [5–9], but ITS could not be amplified from approximately 12% of herbarium samples [3], because ITS1 is too variable to guarantee reliable alignments and contains variable indels (insertions/deletions) at this taxonomic level. Additionally, multiple functional copies exist in many taxa. Thus, ITS was excluded as a universal land plant barcode in the earlier stages. In contrast, ITS2 is considered to have evolved in concert, which leads to a homogenization of all the copies of this gene throughout the genome and in most organisms ITS2 was treated as a single locus. Thus, the ITS2 region might be a suitable marker for taxonomic classification [10–12]. Recently, ITS2 has been suggested as a useful barcode for medicinal plants [13–17], as a universal DNA barcode to identify plants and as a complementary locus of CO1 to identify animals [18]. The China Plant Barcode of Life Group considered ITS2 to be a useful alternative to ITS because it is more easily amplified and sequenced [19]. In addition, the secondary structure of ITS2 was shown to be an efficient tool for biological species identification [20, 21].

 Here, we demonstrated the effectiveness of ITS2 as a minibarcode in comparison with the full-length ITS for the identification of a wide range of archived plant species. An initial set of 100 medicinal samples from museum specimens and the herb market was tested to determine the PCR and sequencing efficiencies of ITS and ITS2. A second set of 12861 sequences, representing 8313 species collected from GenBank, was examined to compare the identification abilities of ITS and ITS2. This work aims to provide an evaluation of ITS2 as a minibarcode for large samples.

## 2. Materials and Methods

### 2.1. Plant Material

**Figure 1:**
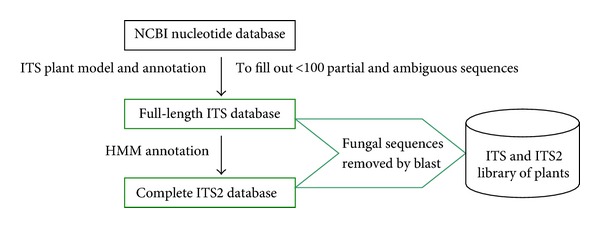
Flowchart of data analysis.

The initial set of 100 museum medicinal specimens and herbal products from 92 species representing 5 orders (see Table 1 of the Supplementary Material available online at http://dx.doi.org/10.1155/2013/741476) was collected from the Buozhou herbal market and from specimens at the Institute of Medicinal Plant Development, some of which were collected 90 years ago, to test the efficiency of PCR and sequencing. All the samples were authenticated at the species level by Professor Yulin Lin (Institute of Medicinal Plant Development, Chinese Academy of Medical Sciences). A second set of sequences for the identification efficiency analysis presented in this paper was obtained from the GenBank nucleotide sequence database. We carried out a bioinformatics analysis using all ITS sequences present in GenBank matching the search pattern “18S ribosomal RNA gene; internal transcribed spacer 1, 5.8S ribosomal RNA gene, and internal transcribed spacer 2, and 28S ribosomal RNA gene.” Partial sequences, fungal sequences, and sequences of less than 100 bp were removed. A flowchart is shown in Figure 1. The complete ITS2 and full-length ITS regions were annotated using the Hidden Markov Model (HMM) [22] and ITS plant model, respectively, which rely on highly similar and correctly annotated reference sequences present in the public database. Ultimately, 12861 sequences representing 8313 species from 1699 genera were obtained (GenBank accession numbers are listed in Table 2S) and used to analyze the identification efficiencies of ITS and ITS2. 

### 2.2. DNA Extraction, PCR Amplification and Sequencing

Total genomic DNA was extracted from specimens using the Plant Genomic DNA Kit (Tiangen Biotech Beijing Co., Ltd., China) according to the manufacturer's instructions. The primer sequences for ITS2 were described by Chen et al. [13]. ITS was amplified using the primers ITS5 and ITS4 [23]. The PCR conditions and sequences used to amplify the two regions (ITS and ITS2) were based on the methods described by Kress et al. and Chen et al. [1, 13, 24, 25].

### 2.3. Analysis Method

Six parameters were used to characterize the interspecific and intraspecific divergences, according to a previously described method [13]. Three of the parameters were used to estimate the interspecific variability: average inter-specific distance, average theta prime, and smallest inter-specific distance. The other three parameters were used to evaluate the intraspecific divergence: average intraspecific difference, theta, and average coalescent depth. The Wilcoxon signed rank test was used as described previously [13, 26, 27]. Basic Local Alignment Search Tool (BLAST1) was performed to identify the species [13]. The TaxonDNA software was used to calculate the identification efficiency [28, 29]. 

## 3. Results 

### 3.1. PCR and Universal Primers

To evaluate the efficiency of PCR and sequencing, 100 medicinal samples from herbal market and museum specimens, including 91 species from 5 orders, were tested; 16% of the samples were obtained from the herb market, and the remaining 84% were obtained from the Institute of Medicinal Plant Development. The ITS primer pair yielded a recovery rate of only 23%, compared with the 91% recovery rate for ITS2. All sequences were submitted to GenBank (the GenBank accession numbers are listed in Table 1S, Supplementary Material). The small size of ITS2 facilitates its amplification by universal primers, even in samples with partially degraded DNA.

### 3.2. Species Identification

#### 3.2.1. Comparison of Inter- and Intraspecific Divergences

Comparison of the inter- and intraspecies sequence variation was an important aspect of the barcoding identification. For the 12861 ITS and ITS2 sequences, which contained 8313 species from 1699 genera, the average lengths of ITS and ITS2 were 634 bp and 233 bp, respectively. The comparison of the inter- and intraspecific genetic distances revealed that the ITS2 region exhibited a higher inter-specific divergence according to the three inter-specific parameters (Table 1). Another advantage of ITS2 is that its conserved secondary structure is associated with relatively low intra-specific variation. The combination of a conserved secondary structure with a variable sequence appears to be a major benefit of using ITS2 [30].

The differences in the percent sequence divergence between loci were tested using the Wilcoxon signed rank test. The results showed that ITS2 was a more variable barcode (Table 2). ITS contained a conserved 5.8S region, which decreased the comparative divergence. Based on these results, ITS2 demonstrates sufficient variation to differentiate plants. 

#### 3.2.2. BLAST-Based Identification

BLAST1 was used to evaluate the efficiencies of ITS2 and ITS. ITS and ITS2 successfully identified 89.2% and 79.2% of specimens, respectively, at the species level and 97.5% and 93.8%, respectively, at the genus level (Table 3). Additionally, the significantly smaller size of ITS2 (average length of approximately 233 bp) compared with that of ITS (average length of approximately 634 bp) makes ITS2 a better candidate for barcoding studies. 

To estimate the respective identification efficiency per genus, genera that contain at least 20 species were selected independently (Table 4). In 85% (68/80) of the genera, the success rates of ITS and ITS2 are identical. ITS had an identification efficiency superior to that of ITS2 in the following 12 genera: *Gunnera*,* Luzula*,* Strobilanthes*,* Nepeta*,* Dionysia*,* Adenia*,* Clidemia*,* Sedum*,* Indigofera*,* Kalanchoe*,* Pilea, *and *Melampodium*. Of the 603 genera that contain at least 3 samples, ITS2 and ITS had the same identification efficiency in 394 genera (65.3%), and ITS and ITS2 shared a 100 % identification efficiency at the species level in 345 genera (57.2%) (Table  3S).

#### 3.2.3. TaxonDNA Identification

We also used TaxonDNA to assess the accuracy of species identification based on ITS and ITS2. TaxonDNA is an alignment-based parametric clustering program that determines the closest match of a sequence by comparing it with all other sequences in the aligned data set. If the compared sequences were from the same species, the identification was considered successful, whereas mismatched names were counted as failures. Cases with several equally good best matches from different species were considered ambiguous [29]. In this study, the successful identification rates of the “best match” were 67.88% and 60% for ITS and ITS2, respectively. The ambiguous identification rates of ITS and ITS2 were 14.9% and 0%, respectively, and the misidentification rates were 17.2% and 40%, respectively. The dataset contained 8607 sequences with duplication.

We used TaxonDNA to set the threshold value. All sequences without a match below the 97% threshold value remained unidentified. If the compared sample names were identical, the identification was considered correct; if the sequence names were mismatched, the identification was considered a failure. When several equally good best matches that belonged to a minimum of two species were found, the identification was considered ambiguous [29, 31]. The successful identification rates under the “best close match” were 62.53% and 32% for ITS and ITS2, respectively. The ambiguous identification rates of ITS and ITS2 were 14.0% and 0%, respectively. The misidentification rates of ITS and ITS2 were 7.28% and 0%, respectively. The remaining samples were considered unidentified because they had no matches below the threshold value. The nonmatch ratios of ITS and ITS2 were 16.2% and 68%, respectively (Table 5). ITS provided slightly superior successful identification and misidentification rates compared with ITS2, but ITS2 provided a lower ambiguous identification rate (0% versus 14.9% and 14.0% under the “best match” and “best close match,” resp., for ITS).

## 4. Discussion

### 4.1. PCR and Sequencing Success Rates

Many museum specimens are very useful for DNA barcoding studies. However, high-quality DNA can be difficult to obtain from these specimens, making PCR amplification and sequencing inefficient. In this study, we recovered short ITS2 sequences from more than 90% of the herbal specimens representing 5 orders, whereas the recovery rate for ITS with a single primer set was only 23%. This discrepancy between the two regions arises because ITS is very long relative to ITS2, and ITS require a variety PCR conditions and additives for successful amplification [32]. Another potential explanation is that intact DNA was difficult to extract from these samples due to the degradation that occurred in the museum specimens during the long storage period and in the herbs from the market during harvesting, processing, and storage. In contrast, the ITS2 region can be easily amplified and sequenced with conserved primers. Due to its relatively short length, the ITS2 minibarcode could be amplified with greater success than the full-length ITS sequences in almost all groups. 

### 4.2. Identification Efficiency of ITS and ITS2

To determine whether barcode gaps are present in this study, the relationships between the inter- and intraspecific divergences were compared for each species. For the 12861 samples, ITS and ITS2 could identify 97.5% and 93.8% of genera, respectively, by the BLAST method. The full-length ITS could identify approximately 89.2% of the species, and the mini-DNA barcode ITS2 successfully identified approximately 79.2% of the species, which is higher than the CBOL proposed plant combination of *matK* and *rbcL *(70%) [4, 5]. 

TaxonDNA was also used to compare the identification efficiencies of ITS and ITS2, and the result appeared to be similar to that obtained by the BLAST method. ITS had slightly superior successful identification and misidentification rates compared with ITS2, but the ambiguous identification rate of ITS2 was 0%, whereas that of ITS was 14.9% and 14.0% under the “best match” and “best close match” algorithms, respectively. The zero ambiguous identification rate of ITS2 may be due to its conserved secondary structure. The secondary structure of ITS2 has proven useful for diagnostic purposes at the species level [21], which might reduce the ambiguous identification rates and increase the correctness of the barcoding analysis. Evidence has shown that a combination of nucleotide and secondary structure data can overcome some of the limitations of ITS2 [33] and that the ITS2 sequence and secondary structure (sequence-structure) provided the most accurate results, which benefit from the secondary structure [30, 34]. Thus, the use of the ITS2 secondary structures would be extremely helpful to address the challenges of species identification and classification.

### 4.3. ITS2 versus ITS: Advantages and Limitations

ITS2 has many advantages that make it superior to ITS. First, it is important that species be defined correctly for DNA barcoding by systematic analysis [3]. ITS2 regions with secondary structures are more conserved than the DNA sequences alone, which could provide information that is useful for the cladistic inference of relationships [35], and the ITS2 sequence-structure information provides a compensatory base changes (CBCs) analysis result that correlates with the biological species concept [21]. Thus, ITS2 has been considered a double-edged tool for evolutionary comparisons in eukaryotes [12].

Second, millions of species will need to be sequenced for a global barcode project, and this would be extremely costly using standard sequencing methods. The read lengths provided by high-throughput sequencing would be sufficient to build a database of ITS2 mini-DNA barcode sequences. High-throughput sequencing technology uses an emulsion PCR approach to simultaneously amplify several thousand 100–200 bp DNA molecules in one reaction and yields a large number of short sequences with a lower cost than standard approaches. Mello proved that the ITS2 read length obtained by high-throughput 454 sequencing provided adequate information for taxon assignment [36]. Song et al. used high-throughout 454 sequencing to successfully obtain a large number of ITS2 sequences in one reaction [37]. The amenability to high-throughput approaches and high identification efficiency makes the ITS2 minibarcode useful for projects involving a large number of environmental samples.

Third, although ITS2 was less powerful than ITS for resolving some closely related species, it showed many advantages, especially in identifying herbs and specimens containing degraded DNA. ITS2 sequences could be used to design taxon-specific probes for the rapid identification of plants [38], and an ITS2 microarray has been used to successfully separate species with sequence identities up to 97% [39]. Considering the short length and high identification efficiency of the ITS2 sequence, we confirmed that this very short barcode sequence is valuable for the identification of old specimens and medicinal materials.

Finally, there are hundreds of copies of ITS within a genome. Nonetheless, ITS2 can be considered a single locus in the whole genome of most organisms [10, 12, 37], including *Panax ginseng *and *Panax quinquefolius *(unpublished), making ITS2 more suitable as a barcode than ITS. 

This study demonstrated the potential of the ITS2 minibarcode for DNA barcoding analyses. ITS2 showed high sequence variability among 12861 samples from 8313 species. An ideal DNA barcoding marker for taxonomic classification should be fast-evolving to allow classification at the species level but must also contain highly conserved priming sites and be highly reliable for DNA amplification and sequencing [40]. The ITS2 region meets the expected criteria of a global DNA barcode. Our analysis supports the use of the ITS2 minibarcode as a “universal DNA barcode” for the rapid identification of medicinal materials and specimens.

## Supplementary Material

Table S1: List of 100 museum medicinal specimens and herbal products from the Buozhou herbal market and from specimens at the Institute of Medicinal Plant Development
Table S2: List of GenBank accession numbers of 12861 ITS sequences
Table S3: List of 603 genera that contain at least 3 samples

## Figures and Tables

**Table 1:** Analysis of interspecific divergence and intraspecific variation of candidate barcodes.

Marker	ITS	ITS2
Avg_intra_avg	0.0145 ± 0.0467	0.0188 ± 0.0792
Avg_intra_max	0.0203 ± 0.0701	0.0327 ± 0.2589
Avg_intra_between_intra-species	0.0308 ± 0.1182	0.0533 ± 0.3202
Avg_interbyG_avg	0.0736 ± 0.0688	0.0959 ± 0.1047
Avg_interbyG_min	0.0329 ± 0.0517	0.0402 ± 0.0719
Avg_between_interbyGenus	0.0752 ± 0.0620	0.1000 ± 0.1138

**Table 2:** Wilcoxon signed rank tests of inter- and intraspecific divergences among loci.

Divergence	Interrelative ranks, *n*, *P* value	Result
Interspecific	*W* + = 2.92*E*8, *W* − = 1.14471579*E*8, *n* = 28495, *P* = 0	ITS2 > ITS
Intraspecific	*W* + = 18423293.50, *W* − = 11658352.50, *n* = 7756, *P* = 6.026542085865164*E* − 66	ITS2 > ITS

**Table 3:** Identification efficiency of ITS and ITS2 by using BLAST.

Marker	Samples	Genus	Species	Length	Identification success at genus level	Identification success at the species level
ITS	12861	1699	8313	633.7	97.5%	89.2%
ITS2	12861	1699	8313	232.6	93.8%	79.2%

**Table 4:** Comparing of the identification rates of ITS with ITS2 in genera with more than 20 species.

			Unidentified species					Unidentified species	
Genus	Species	Samples			Genus	Species	Samples		
			ITS	ITS2				ITS	ITS2
*Acer *	57	341	0.30%	0.30%	*Maxillaria *	245	506	0.20%	0.20%
*Adenia *	37	39	0.00%	2.60%	*Melampodium *	39	93	0.00%	1.10%
*Begonia *	42	71	1.40%	1.40%	*Miconia *	166	175	0.60%	0.60%
*Calceolaria *	64	64	1.60%	1.60%	*Morinda *	20	24	0.00%	0.00%
*Caragana *	28	39	2.60%	2.60%	*Muraltia *	67	67	1.50%	1.50%
*Carex *	73	80	1.30%	1.30%	*Nepeta *	31	31	0.00%	3.20%
*Castilleja *	21	21	4.80%	4.80%	*Nicotiana *	31	43	2.30%	2.30%
*Cineraria *	20	23	4.30%	4.30%	*Oncidium *	76	92	1.10%	1.10%
*Clidemia *	39	40	0.00%	2.50%	*Oxalis *	168	185	0.50%	0.50%
*Cliffortia *	30	33	3.00%	3.00%	*Panicum *	29	39	2.60%	2.60%
*Clusia *	26	63	1.60%	1.60%	*Paphiopedilum *	39	55	1.80%	1.80%
*Coffea *	79	106	0.90%	0.90%	*Pentas *	26	27	3.70%	3.70%
*Costus *	45	75	1.30%	1.30%	*Phyllanthus *	64	82	1.20%	1.20%
*Crinum *	27	27	3.70%	3.70%	*Pilea *	63	66	0.00%	1.50%
*Croton *	45	45	2.20%	2.20%	*Planchonella *	37	42	2.40%	2.40%
*Cuscuta *	44	69	1.40%	1.40%	*Plantago *	68	88	1.10%	1.10%
*Cyrtochilum *	32	42	2.40%	2.40%	*Poa *	44	56	1.80%	1.80%
*Dichaea *	34	58	1.70%	1.70%	*Potentilla *	31	32	3.10%	3.10%
*Dionysia *	35	37	0.00%	2.70%	*Pouteria *	24	27	0.00%	0.00%
*Disterigma *	20	24	4.20%	4.20%	*Rhodiola *	20	47	2.10%	2.10%
*Draba *	122	273	0.40%	0.40%	*Rhus *	21	28	3.60%	3.60%
*Elymus *	35	98	1.00%	1.00%	*Ruellia *	98	116	0.90%	0.90%
*Epimedium *	20	23	4.30%	4.30%	*Salix *	25	33	3.00%	3.00%
*Ficus *	147	152	0.70%	0.70%	*Salvia *	62	124	0.80%	0.80%
*Fritillaria *	38	38	2.60%	2.60%	*Sauropus *	31	37	0.00%	0.00%
*Gagea *	72	141	0.70%	0.70%	*Scaevola *	42	48	2.10%	2.10%
*Garcinia *	43	92	1.10%	1.10%	*Scaphyglottis *	34	41	0.00%	0.00%
*Gomesa *	44	51	2.00%	2.00%	*Sedum *	54	56	0.00%	1.80%
*Gunnera *	20	20	0.00%	5.00%	*Selago *	21	22	4.50%	4.50%
*Hoya *	32	81	1.20%	1.20%	*Senecio *	76	89	1.10%	1.10%
*Indigofera *	52	63	0.00%	1.60%	*Sideroxylon *	42	42	2.40%	2.40%
*Kalanchoe *	49	67	0.00%	1.50%	*Silene *	20	28	3.60%	3.60%
*Kniphofia *	51	96	1.00%	1.00%	*Stevia *	71	91	1.10%	1.10%
*Leandra *	26	26	3.80%	3.80%	*Strobilanthes *	29	29	0.00%	3.40%
*Leontodon *	22	31	3.20%	3.20%	*Swartzia *	22	30	0.00%	0.00%
*Luzula *	25	29	0.00%	3.40%	*Taraxacum *	27	215	0.50%	0.50%
*Macaranga *	38	38	2.60%	2.60%	*Tiquilia *	27	303	0.30%	0.30%
*Macrocarpaea *	46	81	1.20%	1.20%	*Tolumnia *	21	23	4.30%	4.30%
*Mallotus *	29	33	0.00%	0.00%	*Trifolium *	222	257	0.40%	0.40%
*Masdevallia *	34	34	2.90%	2.90%	*Veronica *	132	153	0.70%	0.70%

**Table 5:** Identification success based on “best match” and “best close match.”

	Best match		Best close match	
	ITS	ITS2	ITS	ITS2
Correct identification (%)	67.88	60	62.53	32.00
Ambiguous identification (%)	15	0	14.0	0.00
Incorrect identification (%)	17	40	7.28	0.00
Without any match closer than 3.0% (%)	—	—	16.20	68.00
